# Molecular and Serological Identification of *Anaplasma marginale* and *Borrelia burgdorferi* in Cattle and Ticks from Nuevo Leon, Northern Mexico

**DOI:** 10.3390/pathogens12060784

**Published:** 2023-05-31

**Authors:** José Ángel Ortiz-Ramírez, Jorge Jesús Rodríguez-Rojas, Jesús Jaime Hernández-Escareño, Kame-A Galan-Huerta, Eduardo Alfonso Rebollar-Téllez, Gustavo Moreno-Degollado, Carlos E. Medina-De la Garza, Rosa María Sánchez-Casas, Ildefonso Fernández-Salas

**Affiliations:** 1 Facultad de Medicina Veterinaria y Zootecnia, Universidad Autónoma de Nuevo León, Escobedo 66054, NL, Mexico; 2 Unidad de Patógenos y Vectores, Centro de Investigación y Desarrollo en Ciencias de la Salud, Universidad Autónoma de Nuevo León, Monterrey 66460, NL, Mexico; 3 Departamento de Bioquímicay Medicina Molecular, Facultad de Medicina, Universidad Autónoma de Nuevo León, Monterrey 66460, NL, Mexico; 4 Facultad de Ciencias Biológicas Laboratorio de Entomología Médica, Universidad Autónoma de Nuevo León, San Nicolás de los Garza 66455, NL, Mexico

**Keywords:** bovine anaplasmosis, Lyme disease, *Rhipicephalus microplus*, tick-borne disease

## Abstract

Ticks and tick-borne diseases affect livestock productivity and cause significant economic losses. Therefore, surveillance of these pathogens and vectors is paramount to reducing these effects in livestock. This study aimed to identify *Anaplasma marginale* and *Borrelia burgdorferi* sensu lato in ticks collected from cattle. Molecular biology techniques were utilized to identify *A. marginale* for both types of samples, i.e., ticks and bovine blood. Serology of cattle using indirect immunofluorescence assay (IFA) was conducted to determine antibodies to *B*. *burgdorferi* s.l. from seven locations in Nuevo Leon, Mexico, between 2015 and 2017. From 404 bovines, 2880 ticks were collected: *Rhipicephalus microplus* (2391 females and 395 males), *Amblyomma* spp. (51 females and 42 males) and *Dermacentor variabilis* (1 female). *Rhipicephalus microplus* represented the largest specimens captured, with 96.7% within the seven study sites. PCR processed only 15% (442) of tick samples to identify *A. marginale*. Field genera proportions were followed to select testing tick numbers. Results showed that 9.9% (44/442) of *A. maginale* infected the pooled tick species, whereas the highest percent corresponded to 9.4% (38/404) in *R. microplus*. Regarding the molecular analysis of blood samples, 214 of 337 (63.5%) were positive for *A. maginale*. In each of the seven locations, at least one bovine sample tested positive for *A. maginale*. *Borrelia burgdorferi* s.l. was not found either in the ticks or serum samples. Two *A.marginale* DNA nucleotide sequences obtained in this study were deposited in the GenBank with the following accession numbers OR050501 cattle, and OR050500 *R.microplus* tick. Results of this work point to current distribution of bovine anaplasmosis in northern Mexico.

## 1. Introduction

*Anaplasma marginale* Theiler 1910 (Rickettsiales: Anaplasmataceae) is an obligate intracellular Gram-negative bacterium that parasites eukaryotic cells of only ruminants and causes bovine anaplasmosis. The most characteristic symptoms are fever, severe anemia, hemolytic anemia pyrexia, depression, abortion, decreased milk production, and weight loss. Bovine anaplasmosis is a disease that occurs in tropical and subtropical areas that impacts cattle production in many countries [1,2]. *Anaplasma marginale* is transmitted mainly by *Rhipicephalus* ticks but can also be transmitted mechanically by biting flies, blood-contaminated needles, contaminated farm equipment, and transplacental transmission [1]. At the same time, *Borrelia burgdorferi* Johnson, Schmid, Hyde, Steigerwalt, and Brenner 1984 (Spirocheatales: Spirochaetaceae) is a Gram-negative spirochete bacterium, a motile, microaerophilic, and helical-shaped microorganism that is the causative agent of borreliosis or Lyme disease [3]. This disease can affect domestic and wild animals and infect humans. In cattle, acute Lyme borreliosis often will show a swollen joint, stiffness, fever, decreased milk production, and to a lesser extent, laminitis, chronic weight loss, and abortion [4]. This species of bacteria is transmitted mainly by ticks of the *Ixodes* genus. Moreover, *Dermacentor variabilis* (Say 1821) or *Amblyomma americanum* (Linnaeus 1758) play an essential role in the transmission to livestock [5].

Bovine anaplasmosis is present in several southern, central, and northern Mexico states, including Nuevo Leon. The prevalence of *A*. *marginale* in cattle from different regions of Mexico ranges from 11 to 70% [6,7]. Moreover, *A*. *marginale* has been found in *R*. *microplus* (Canestrini 1888) from Tamaulipas [8]. While the information on *B*. *burgdorferi* in cattle is almost nil, only one report found *Amblyomma cajennense* sensu lato (Fabricius 1787) nymphs captured in three cattle, of which two ticks were positive for *B*. *burgdorferi* sensu stricto in Mexico [9]. There is more evidence in Mexico of the natural infection of *B*. *burgdorferi* in ticks since it has been reported in about thirteen species [5].

At the national level, it is estimated that 1,097,930 production units are dedicated to the breeding and exploitation of cattle, according to the National Agricultural Survey of Mexico [10]. All age groups of cattle populations are susceptible to bovine anaplasmosis and borreliosis. And this risk increases because Mexico is an endemic region for these diseases. Therefore, current and specific studies are needed on monitoring *A*. *marginale* and *B*. *burgdorferi* in cattle and their associated ticks. Thus, the present study aims to identify these two bacteria in cattle blood samples and ticks by molecular biology. Only *B. burgdorfei* antibodies testing was conducted by the IFA technique from seven locations in Nuevo Leon, Mexico.

## 2. Materials and Methods

### 2.1. Study Areas

**Figure 1:**
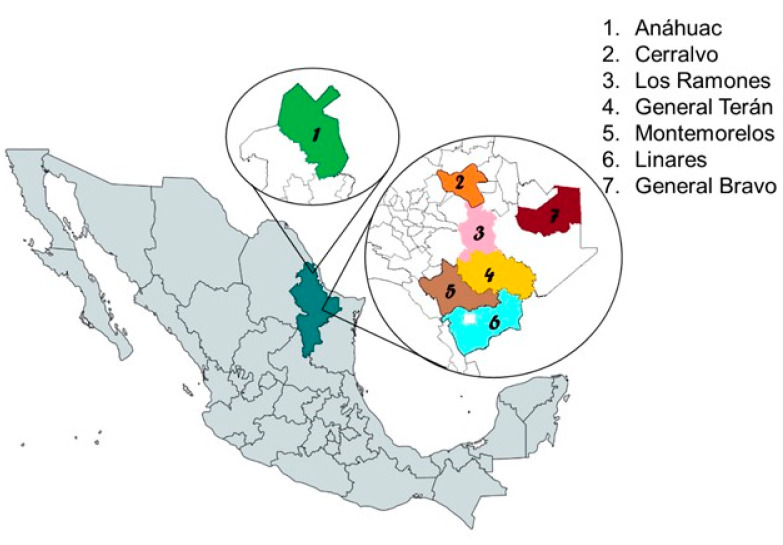
Geographic location of the seven study sites of molecular surveillance of *Anaplasma marginale* and *Borrelia burgdorferi* in cattle and ticks from Nuevo Leon, Mexico, between 2015 and 2017.

The study was conducted in seven municipalities of Nuevo Leon, Mexico: 1. Anahuac (27°14′27.5″ N, 100°08′01.5″ W; altitude 335 m), 2: General Teran (25°15′34.0″ N, 99°40′50.7″ W; altitude 230 m), 3. Los Ramones (25°41′47.0″ N, 99°37′27.9″ W; altitude 226 m), 4. General Teran (25°15′34.0″ N, 99°40′50.7″ W; altitude 230 m), 5. Montemorelos (25°11′13.0″ N, 99°50′05.0″ W; altitude 342 m), 6. Linares (25°51′45.2″ N, 99°33′52.0″ W; altitude 350 m), and 7. General Bravo (25°47′44.2″ N, 99°10′44.3″ W; altitude 150 m) (Figure 1). These study areas belong to the physiographic region of the North Gulf Coastal Plain, and the Tamaulipas Spiny Scrub ecoregion. The altitude of these sites ranges between 150 and 350 m. The climate is dry and semidry. The average annual temperature is around 20 °C, while the average yearly rainfall is 650 mm, with summer rains in August and September [11]. The sampling was conducted from 2015 to 2017, in April, June, July, September, October, and November.

### 2.2. Ticks and Bovine Blood Collections

Every herd of each locality was privately owned, and it was worked with the owner’s approval. Ethics committee approval for our study was number CEICANCL21012022- MOLSUNL-06. Cattle were manually restrained in a Powder River manual squeeze chute (crush). Ticks were captured directly from cattle (Simbrah and Simmental breed) and placed in vials with 90% alcohol.

Each tick was carefully observed under 8 to 32× magnifications using a stereoscopic microscope (Carl Zeiss Light Microscopy, Göttingen, Germany) to examine the external morphological characteristics of genera and species; in this way, the life stage and sex of each specimen were also determined. The identification was based on the taxonomic keys of [12,13,14,15]. Voucher tick specimens are deposited in the Walter Reed Biosystematics Unit, Smithsonian Institution, Suitland, MD, USA.

In contrast, the whole blood samples for molecular assay were taken from the caudal vein of the bovine in sterile tubes with EDTA, one sampling per animal. The tubes were immediately transported to the laboratory on ice (4 °C). Separate blood samples were allowed to settle and centrifuged at 2000× *g* for 10 min. The serum was recovered and stored at −20 °C until *B. burgoderferi* serological analyses.

### 2.3. DNA Extraction and PCR Amplification

DNA extraction from both ticks and whole blood was performed using the method of Ferrer et al. [16]. From the material obtained after crushing the tick in a mortar, the entire content was added to a new Eppendorf tube, and 500 μL of lysis buffer and 40 μL of proteinase K were placed and left to incubate in an AccuBlock (Labnet International Inc., Edison, NJ, USA) dry bath at 65 °C for one hour, shaking the sample in a vortex (VX-100 Lab Vortex Mixer Labnet International Inc.) every 15 min. After one hour, 500 μL of phenol–chloroform–isoamyl alcohol (49.5:49.5:1) was added to the sample and placed in a centrifuge (Centrifuge 5430 Eppendorf) at 14,000 rpm for 15 min. The supernatant was placed in a new tube, and two washes were performed with 500 μL of isoamyl alcohol (27:1), centrifuging for 10 min at 14,000 rpm, and collecting the supernatant. Subsequently, 65 μL of NaOAc was added, and 75 μL of NaCl was gently shaken using inversion. It was left at −20 °C for 24 h. The sample was centrifuged for 10 min at 14,000 rpm. The supernatant was collected and placed in a new tube to which 270 μL of isopropanol was added and left at −20 °C for 10 min. The sample was centrifuged for 10 min at 14,000 rpm, and the supernatant was removed. The pellet was resuspended in 500 μL of 80% molecular-grade ethanol and centrifuged for 5 min at 14,000 rpm. Once centrifuged, the supernatant was removed and placed in the 37 °C incubator to dry the ethanol completely. The DNA was resuspended in 20 μL of milli-Q water or TE buffer, and the DNA was extracted at −20 °C to continue with PCR.

For *B. burgdorferi*, the 234 bp PB directed at the flagellin gene sequence SC1 (5′-AAC ACA CCA GCA TCA CTT TCA GG-3′) and SC2 (5′-GAG AAT TAA CTC CGC CTT GAG AAG-3′) [17] was used. With the denaturation cycle at 94 °C for 1 min, 40 denaturation cycles at 93 °C for 30 s, primer binding at 57 °C for 30 s, DNA chain elongation at 72 °C for 30 s, and finally 72 °C for 1 min of elongation. Once the reaction is complete, the fraction is observed on an agarose gel.

For *A. marginale*, the 265 bp (Msp1 β), which is directed to the surface protein sequence SC1 (5′-GCT CTA GCA GGT TAT GCG TC-3′) and SC2 (5′-CTG CTT GGG AGA ATG CAC CT-3′ [18], was used with the denaturation cycle at 94 °C for 1 min, 30 denaturation cycles at 95 °C for 1 min, primer binding at 53 °C for 1 min, DNA chain elongation at 72 °C for 1 min, and finally at 72 °C for 10 min of elongation. Once the reaction was complete, the fraction was observed; all PCR products of ticks and bovine blood were carried out by electrophoresis on 1% agarose gels. They were visualized with ethidium bromide staining under ultraviolet light. Amplicons from blood cattle and the vector *R. microplus* were used for *A. marginale* sequencing, and further deposited to the GenBank.

### 2.4. Serological Analysis

Considering the almost unknown Lyme borreliosis in cattle in the area, we focused our study on only evaluating the presence of antibodies against *B*. *burgdorferi* using IFA. The substrate and bovine-specific anti-immunoglobulin to perform the tests were purchased commercially (Lyme (*B*. *burgdorferi*) IFA Substrate Slide from VMRD^®^ laboratory; bovine anti-immunoglobulin). Of the serum, ten μL with titers of 1:64 was used, with an incubation time of one hour at 37 °C in a humid chamber. After the anti-immunoglobulin was conjugated with fluorescein isothiocyanate (FITC), ten μL was used, incubating at 37 °C for 45 min.

### 2.5. Statistical Analysis

The sample size of ticks for molecular analysis was calculated with a formula for a finite population with a 95% confidence level and a margin of error of 5%. Statistical analyses for variable associations were performed using the chi-square test. Infection rates were compared by study site and positive molecular samples. A value of *p* < 0.05 was considered significant. All analyses were conducted using the Epi InfoTM program ver. 7.2 from Centers for Disease Control and Prevention, Atlanta GA USA [19].

## 3. Results

### 3.1. Ticks Collected from Cattle

The group of 404 bovines yielded 2880 ticks distributed in three genera: *R*. *microplus* (2391 females and 395 males), *Amblyomma* spp. (51 females and 42 males), and *D*. *variabilis* (1 female). *Rhipicephalus microplus* represented the most significant number of specimens captured, with 96.7%. Within the seven study sites, Linares captured the highest number of ticks (29.9%), followed by General Teran (22.9%) (Table 1).

### 3.2. Ticks Sample Size from Cattle for PCR Analysis

Several 442-ticks, representing 15% of the total population of 2880 collected were calculated for molecular PCR analysis. The number of individuals per tick species described the same proportions as in the field frequency: *R*. *microplus* (n = 404), *Amblyomma mixtum* Koch 1844 (n = 10), *A*. *tenellum* (n = 27), and *D*. *variabilis* (n = 1).

### 3.3. Ticks Analyzed Using PCR to Identify A. marginale

*Anaplasma marginale* infection rates varied by tick species and study sites. Our results showed that, from 442 assayed individuals, only 44 (9.9%) ticks were positive for *A*. *marginale*. The number of positive specimens per species was *R*. *microplus* (n = 38; 9.4%), *A*. *tenellum* (n = 5; 18.5%), and *D*. *variabilis* (n = 1; 100%). Likewise, the localities with the highest infection rates of *A*. *marginale* in ticks were Linares (19.8%) and Montemorelos (18.7%) (Table 2). There was a significant (χ^2^ (4, n = 428) = 28.83, *p* < 0.00) association between study sites (Linares, General Teran, General Bravo, Cerralvo, and Montemorelos) and infection rate of *A*. *marginale* in ticks. Moreover, there was a significant (χ^2^ (4, n = 392) = 23.71, *p* = 0.00) result between localities (Linares, General Teran, General Bravo, Cerralvo, and Montemorelos) and the infection rate of *A*. *marginale* in *R*. *microplus*. One amplicon of *R. microplus* infected with *A. marginale* was sequenced and uploaded to the GenBank as a molecular genetic reference; the accession number is OR05500.

### 3.4. Bovine Blood Samples Analyzed Using PCR to Identify A. maginale

Of the 404 bovines, 337 blood samples were PCR-processed and yielded 214 positives for the presence of *A*. *maginale* (65.50%) infection rate (Table 3). Rates of cattle infected with *A*. *marginale* showed variation among localities. Cerralvo showed the highest infection rate of 92.45%, followed by Montemorelos (81.82%). A chi-square test examined the association between study sites (Linares, General Teran, General Bravo, Cerralvo, and Montemorelos) and infection-positive *A*. *marginale* in cattle. The relation between these variables was significant, χ^2^ (4, n = 327) = 20.02, *p* = 0.000. There was a significant association between study sites and the *A*. *marginale* infection rate. A host or cattle sample showing PCR infection to *A. marginale* was sequenced and GenBank accessed, number OR050501.

### 3.5. Ticks and Blood Bovine Samples Negative to B. burgdorferi s.l. by PCR and IFA

Negative results were determined using IFA antibody serology to identify *B*. *burgdorferi* s.l.; there was no identification in blood samples (n = 367) from cattle or PCR testing on ticks (n = 442). The same result was consistent throughout all study sites.

## 4. Discussion

In this work, the endpoint PCR identified *A*. *marginale* in cattle blood and tick tissue from several Nuevo Leon, Mexico localities. The amplification of the Msp1β gene delivered a product of the expected molecular size of 265 bp, confirming the presence of this hemoparasite in cattle and ticks. These data represent the first report in Nuevo Leon.

Bovine anaplasmosis is reported to be endemic in cattle in Mexico [7]. The infection rates of *A*. *marginale* in cattle in Mexico range from 10 to 78.9% [20,21,22]. The present study found an average infection rate of 63.50% in bovines in all seven sampled localities of Nuevo Leon. Percent rates ranged from 20% at Anahuac and Los Ramones to 81.82% in Montemorelos and 92.45% in Cerralvo. In other studies, a variation in the infection rates was found, for example, in the neighboring state of Tamaulipas by Lopez-Sanchez et al. [23], reporting 11.1% using serology, and by Almazán et al. [8] 36.7% using blood smear. Moreover, other percentages of infection of *A*. *marginale* in cattle from some southern states of Mexico have been documented. Pacific Ocean coastal states such as Guerrero (78.9% serology) [24], Veracruz (56% serology and 69.2% PCR) [25,26], and Yucatan (69.8% serology) [21] showed the highest percentages of infection. These differences between the data may be due to different ecological conditions; the breed and age of cattle; the difference in sample size and sampling effort of each locality; and the high infestation of susceptible tick populations.

Molecular identifications exhibited 9.9% of infection found in all the tick species and study localities (Table 2). Five of the seven localities resulted in favorable rates, whereby localities such as Montemorelos and Linares showed the highest rates: 18.7% and 19.8%, respectively. Although *A*. *marginale* was found in Anahuac and Los Ramones cattle, their tick populations were PCR-negative. The number of ticks captured in Anahuac (n = 10) and Los Ramones (n = 3) was very low compared to the other localities. Thus, the probability decreases in the detection of the bacteria. Conversely, Montemorelos and Cerralvo were the opposite. Particularly in Montemorelos, where both reservoir and vector yielded (81.82%) and (18.7%) infected with the bacteria, respectively.

In Mexico, it has been documented that two tick species, *R*. *microplus* and *Rhipicephalus annulatus* (Say, 1821), are proven vectors of *A*. *marginale* [2,27]. Only one study from Tamaulipas State has demonstrated 40% of the natural infection of *A*. *marginale* in *R*. *microplus* [8]. Here, we were first to reveal infection of *A*. *marginale* in *R*. *microplus* collected from cattle from Nuevo Leon. Similarly, we reported the presence of *A*. *marginale* in the ticks *A*. *tenellum* and *D*. *variabilis*. Our results showed that *A*. *marginale* in *R*. *microplus* and *A*. *tenellum* played a variable role as vectors from locality to locality. This result suggests that *R*. *microplus* may act as a vector of *A*. *marginale* in Linares, General Teran, General Bravo, Cerralvo, and Montemorelos. Undoubtedly, our findings point out that *Rhipicephalus microplus*, known as cattle tick, is a species that has a wide distribution in the American region and causes severe damage to cattle due to its direct parasitic action and because it is a vector of important pathogens [28].

We have shown here the natural infection of *A*. *marginale* in cattle and its *R*. *microplus*, *A*. *tenellum,* and *D*. *variabilis* ticks from some Nuevo Leon, Mexico localities. Surveillance is the first step to controlling tick-borne diseases; it is necessary to monitor ectoparasites and their causal agents [29]. This is important to avoid economic losses in Mexico, with a cattle population of 35,224,960 heads at risk [6]. Therefore, this work offers a clearer perspective on bovine anaplasmosis in Nuevo Leon for the first time. 

In addition to surveillance, accurate diagnostic methods are the next step to confirming specific pathogen presence. Several traditional and modern approaches have been developed: microscopic observation, cELISA, card agglutination test, and, more recently, PCR [1]. However, the PCR method used in the present study has gained more popularity due to its specificity. Thus, it can be used in epidemiological studies as an early and accurate diagnostic method [30].

Although this study showed negative results for *B*. *burgdorferi* in cattle and ticks from Nuevo Leon, in a systematic review, a total of 1,347 records of *Borrelia* were found in Mexico [5]. Additionally, Nuevo Leon was one of the three states with the highest number of reports (253 records; 18.5%): humans [31,32,33,34], domestic dogs [35,36], wild rodents [37], and ticks [9,38]. However, there are no reports of this spirochete in cattle in Nuevo Leon.

For perspective and future studies of these diseases, it will be necessary to sequence the positive samples according to collection localities, tick species, and disease incidence, to carry out the respective phylogenetic and clinical analyses. Since it is known that there is a genetic diversity of *A*. *marginale* in Mexico [8,39,40], this way establishes superinfections [20] in places with a high rate of infection such as Montemorelos and Cerralvo, Nuevo Leon, Mexico. Therefore, molecular surveillance of these two diseases must be continued.

## 5. Conclusions

In the current study, we made the first presentation of *A*. *marginale* natural infection in the following tick species: *R*. *microplus*, *A*. *tenellum,* and *D*. *variabilis*. These species were collected from domestic cattle from Nuevo Leon, northeastern Mexico. Molecular PCR techniques confirmed *A*. *marginale* in bovine blood samples. Conversely, we did not find *B*. *burgdorferi* in the same ticks and bovine blood samples. 

## Figures and Tables

**Table 1:** Species of ticks captured from bovines in seven localities of Nuevo Leon, Mexico, from 2015 to 2017.

Localities	*Rhipicephalus* *microplus* (F:M)	*Amblyomma* spp. (F:M)	*Dermacentor* *variabilis* (F:M)	Total (F:M)
Linares	860 (734:126)	1 (0:1)	1 (1:0)	862 (735:127)
General Teran	653 (584:69)	7 (7:0)	0	660 (591:69)
General Bravo	256 (182:74)	21 (3:18)	0	277 (185:92)
Cerralvo	523 (469:54)	10 (8:2)	0	533 (477:56)
Montemorelos	355 (297:58)	53 (32:21)	0	408 (329:79)
Anahuac	134 (120:14)	0	0	134 (120:14)
Los Ramones	5 (5:0)	1 (1:0)	0	6 (6:0)
Total (F:M)	2786 (2391:395)	93 (51:42)	1 (1:0)	2880 (2443:437)

Note. F: female; M: male

**Table 2:** *Anaplasma marginale* infection rates (%) in ticks collected from cattle at study sites in Nuevo Leon, Mexico, from 2015 to 2017.

Localities	*Rhipicephalus microplus* (%)	*Amblyomma mixtum* (%)	*Amblyomma tenellum* (%)	*Dermacentor variabilis* (%)	Total (%)
Linares	20/105 (9.5)	0	0	1/1 (100)	21/106 (19.8)
General Teran	5/110 (4.5)	0	0/4 (0)	0	5/114 (4.4)
General Bravo	3/60 (5.0)	0/4 (0)	0	0	3/64 (4.7)
Cerralvo	1/65 (1.5)	0/5 (0)	0	0	1/70 (1.4)
Montemorelos	9/52 (17.3)	0	5/23 (21.7)	0	14/75 (18.7)
Anahuac	0/10 (0)	0	0	0	0/10 (0)
Los Ramones	0/2 (0)	0/1 (0)	0	0	0/3 (0)
Total (%)	38/404 (9.4)	0/10 (0)	5/27 (18.5)	1/1 (100)	44/442 (9.9)

**Table 3:** Positive PCR blood samples of *Anaplasma marginale* from cattle at study sites in Nuevo Leon, Mexico from 2015 to 2017.

Localities	Total Samples	+ Samples	Total (%)
Linares	95	61	64.21
General Teran	90	34	37.78
General Bravo	45	32	71.11
Cerralvo	53	49	92.45
Montemorelos	44	36	81.82
Anahuac	5	1	20.00
Los Ramones	5	1	20.00
Total	337	214	63.50

## Data Availability

Data is private property of cattle farm owners.
